# Nucleation of cadherin clusters on cell-cell interfaces

**DOI:** 10.1038/s41598-022-23220-x

**Published:** 2022-11-02

**Authors:** Neil Ibata, Eugene M. Terentjev

**Affiliations:** Cavendish Laboratory, University of Cambridge, JJ Thomson Avenue, Cambridge, CB3 0HE UK

**Keywords:** Biological physics, Statistical physics, thermodynamics and nonlinear dynamics, Biophysics

## Abstract

Cadherins mediate cell-cell adhesion and help the cell determine its shape and function. Here we study collective cadherin organization and interactions within cell-cell contact areas, and find the cadherin density at which a ‘gas-liquid’ phase transition occurs, when cadherin monomers begin to aggregate into dense clusters. We use a 2D lattice model of a cell-cell contact area, and coarse-grain to the continuous number density of cadherin to map the model onto the Cahn-Hilliard coarsening theory. This predicts the density required for nucleation, the characteristic length scale of the process, and the number density of clusters. The analytical predictions of the model are in good agreement with experimental observations of cadherin clustering in epithelial tissues.

## Introduction

Many eukaryotic cells use membrane-bound integrin adhesion clusters^1^ to tether themselves to the extra-cellular matrix surrounding them (ECM). Similarly, these cells use membrane-bound cadherin adhesion clusters^2–4^ to bind to their neighbouring cells directly. Adhesion molecules mediate mechanical signalling between the cell and its exterior by participating in important intracellular signalling pathways^5–8^. Clusters of adhesion molecules also help determine the structure of the cell; these shape changes are essential if the cell is to topologically fit into a tissue (e.g. in dividing epithelia^9^), to change its function (fibroblasts differentiating into myofibroblasts when placed on stiff media^10,11^), or to move (during wound healing^12,13^ or cancer metastasis^14,15^). In order to understand why any of these processes occur, we must first understand why there are clusters of adhesion molecules at all – in physical terms, how their nucleation from a uniform distribution of sensors occurs.

Density-dependent nucleation is ubiquitous in soft matter. Changes in the concentration of attractively coupled molecules can help form large-scale symmetry-breaking structures. Computational studies have investigated the clustering of both integrin^16,17^ and cadherin^18,19^. The classical theory of aggregation on fluctuating membranes^20–22^ explains the unstable growth of nuclei into large-scale receptor domains (evident in low-resolution experiments), notably including the case of large cadherin domains on vesicles^23^. These effects are driven by the weak long-range forces mediated by membrane fluctuations, and cannot account for the stability of small nanometer-scale clusters of the kind recently identified in high-resolution cadherin imaging^24,25^. We recently analytically investigated the nucleation of integrins in the high-concentration limit on the edge of a spreading cell^26^. Here, we extend this approach to the nucleation of such small punctate cadherin clusters on a generic cell-cell contact surface, stabilized by the strong short-range bonding between monomers.

We will first review background literature to examine the parallels between the aggregation mechanisms for these two adhesion molecules. Next, we build a lattice gas model to obtain the Ginzburg-Landau free energy for fluctuations in the density of cadherin molecules on the realistic 2D contact plane between cells. We consider whether our model correctly predicts the size and spacing of punctate adherens junctions seen in experiments.

### Integrin/Cadherin analogy

**Figure 1:**
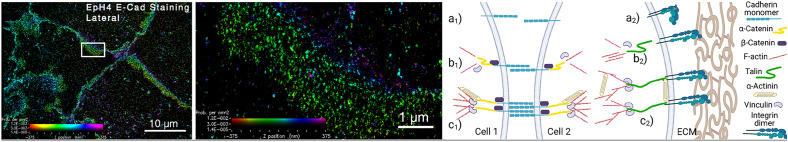
Left panel: E-cadherin (‘E’ standing for epithelial) aggregate into punctate clusters with a characteristic size $\lesssim 50$ nm and separation $\gtrsim 100$ nm. Reproduced with permission from Wu et al.^24^. Right panel: Aggregation of cadherins (1) and integrins (2). (**a**) Single adhesion molecules sit in the cell membrane, inactive. (**b**$_1$) Catenins begin to aggregate around the transmembrane cadherins, which form transmembrane *trans* bonds, and link to the cytoskeleton^3,42^: an individual adhesion complex forms. (**b**$_2$) Integrins are activated by pulling when an individual adhesion complex assembles^43^. (**c**) Adhesion units aggregate laterally via cadherin *cis* bonds (1) or auxiliary proteins for integrin (2), and form clusters.

The cell needs to develop adhesion clusters in order to correctly remodel its shape in response to external substrates and mechanical cues^27,28^. The large clusters visible under the microscope (called focal adhesions for integrins^10,29^, and zonula adherens for cadherin^30,31^) are the end-product of many smaller clusters aggregating over minutes or hours. In order to be stabilized, both integrins^32^ and cadherins^33^ rely on ‘catch bonds’^34^, which strengthen under load. These bonds can help activate both molecules and are often preceded by the formation of a larger cytoplasmic protein complex which links an individual adhesion molecule to the cytoskeleton (see Fig. 1). Integrin uses talin and vinculin to bind to F-actin^35,36^, while cadherin primarily relies on catenins^37,38^. After the protein complex is fully assembled inside the call, the acto-myosin cytoskeleton exerts a pulling force on the adhesion molecule, strengthening the catch bond. Individual integrin complexes can then aggregate into growing clusters, and link the cytoskeleton with the outside of the cell over a larger area, spreading the force applied by the actomyosin cortex^39,40^. Provided that adhesion molecules cluster together in sufficient numbers to withstand the load, the cytoskeleton can develop increasingly large pulling forces and distort the shape of the cell^41^. ‘

Cadherins differ most notably from integrins in their size and ability to multimerise. Whereas integrins appear to only use secondary molecules to link to each other (e.g. $\alpha$-actinin^44–47^), cadherins can form two types of bonds directly with each other. *Trans* bonds link cadherins from two different cells^48,49^, and effectively mimic the integrin binding to extra-cellular matrix; *cis* bonds link neighbouring cadherins of the same cell in the plane of the membrane.

Their cytoplasmic force chain uses $\alpha$- and $\beta$-catenin instead of talin and vinculin to connect to the actin cytoskeleton. The $\alpha$-catenin-actin bond is notable as it also strengthens as a catch bond, but as it only supports substantially lower forces of up to 8 pN, it seems likely that the cadherin-catenin complexes often detach from their cytoskeletal contacts^50^. And although the absence of $\alpha$-catenin causes ultrastructural disruption to cells (*e.g.* cardiac intercalated disc specific $\alpha$-E-catenin suppression disrupts cardiomyocyte structure in mice and predisposes them to heart attacks^51^, $\alpha$-E-catenin and $\alpha$-T-catenin double knockout mice possess less well organised intercalated disks ?), N-cadherin clusters are still present at the intercalated disk after $\alpha$-E-catenin/$\alpha$-T-catenin depletion?. This means that unlike in integrin complexes, cytoskeleton-cadherin links are not essential for adherens junctions to form and to be maintained.

The distance between cadherin monomers in a loosely packed cadherin lattice is ca.7 nm^52–54^, but can be as small as 3 nm in very tightly packed junctions^55^; both of these figures are much smaller than the separation between neighbouring integrins: ca.30 nm^56^. The *cis* bond strength can be estimated by extrapolating from measurements of cadherin *trans* bond strength and the dissociation rates of these bonds, as well as from numerical simulations of cadherin clustering. Note that the cadherin dissociation rate is much faster in loose cadherin clusters than in well-developed adhesion junctions with many such bonds established^57^.

### Cadherin-cadherin bonds

Whether the cadherin *trans* or *cis* bonds are most important to the formation of adherens junctions has long been the topic of debate in the field. Over the last ten years, the classical view has shifted from the idea that *cis* dimerisation preceded *trans* dimerisation^58^. The discovery of an intermediate crossed-dimer, called the X-dimer, which precedes a more stable swapped *trans* dimerisation suggests that *cis* dimerisation is not necessary for cadherins to form stable cross-membrane bonds^52,59^. More recent work^60^ suggests that cadherin cross-membrane dimers form before *cis* clustering occurs. This can be further quantified by examining recent work on the development of large adherent regions in giant unilamellar vesicles (GUVs). In particular, Fenz et al.^23^ showed that cadherin *trans* bonding, a characteristic of the extended adhesive region, spread from nucleation sites if membrane fluctuations were sufficiently small. In Supplementary Part A, we show that the size of membrane fluctuations within an adhesive region in a cell is much smaller than that required for adhesive regions to separate into separate domains in this model, based on the data from^61,62^. This means that within an adhesive region, a large portion of cadherins are indeed within *trans* bonded dimers (with *on*-rates $\ll 1$ s, given an intrinsic lifetime of 0.63 s^33^). Lateral interactions between cadherin *trans* bonded pairs are small in this model (a few $k_B T$ at most), relying on membrane fluctuations to slowly and randomly bring cadherin pairs together. In contrast, small crystalline clusters of cadherin have been recently found^24,25^ (see Fig. 1), and these disappear if the *cis* abolishing V81D/V175D mutation is introduced^52^. This means that while cadherin *trans* interaction mediated by membrane fluctuations does lead to the development of the larger adhesive area, it is insufficient to help develop individual punctate cadherin adhesions.

Cadherin *trans* bond strength has been suggested to be in the range 9–$13 k_B T$, substantially greater than the $J\le 7 k_B T$ suggested for *cis* bond^24^. The *on*-rates of *trans* bonds should therefore be 10–100 times greater than those of *cis* bonds, and we expect, as in much of the literature, for *trans* bonds to form before *cis* bonds. The problem would then no longer need to be resolved separately in both cells; rather, we need only look at the *cis* (in plane) clustering of the cadherin *trans* dimers on the adhesion surface. Recent computational work by Yu et al.^63^ (see their Fig. 3B) shows that there is an optimum cis-bond strength for cluster formation of 3–$8 k_B T$. We take $5 k_B T$ as a compromise value, given previous research suggesting that *cis* bond strength is smaller than *trans*^59^.

Purified E-cadherin molecules form clusters on vesicle membranes *in vitro*^19^ without *trans* bonds or forming cytoplasmic complexes with other molecules. They form clusters of 30 or so cadherins above a relatively low critical density of 1100 cadherins $\mu \text {m}^{-2}$, much lower than the average cadherin concentration observed by Wu et al.^24^. Cadherin-cadherin *cis* bonds clearly lead to clustering *in vitro*, and they appear to be necessary for the formation of small clusters that diffuse in the absence of cytoskeletal linkers? as well as the stabilization of larger intercellular junctions? *in vivo*.

### Cadherin-cadherin interactions in vivo

Super-resolution microscopy by Wu et al.^24^ brought an additional complication to the problem, as they found that punctate adhesions could form small clusters without either *cis* or *trans* interactions. The effect of cadherin-cadherin bonding was to make the core of the punctate adhesions denser, rather than to alter the number of cadherins per cluster or the distance between clusters. They suggested that the cytoskeleton might form actin fences around the cadherins and force them into clusters in that manner. This hypothesis seems problematic as some of the cadherin clusters that they found were not surrounded by actin (see the top of Panel F or the bottom of Panel I in their Figure 5), and because adherens junctions can form without cytoskeletal links (see discussion above^51^). If cadherin adherens junctions can form without cadherin-cadherin bonds, $\alpha$-catenin, or cytoskeletal forcing, then the only candidates remaining for direct lateral interactions between cadherins are $\beta$-catenin (a key component of the adherens junction^64^) or molecules that bind to it. $\beta$-catenin knockout is immediately fatal to embryos soon after gastrulation, showing detached ectodermal cells floating in the proamniotic cavity^65^. This strongly suggests that $\beta$-catenin plays an essential role in the initial formation of junctions in the embryo as well as in their later cycling and maintenance. However, $\beta$-catenin is asymmetric and can only bind one cadherin molecule, so it cannot cause an attractive interaction between two neighbouring cadherins.

The structure of the cadherin cytoplasmic complex is still not fully elucidated, so it is impossible to rule out other candidates, but we suggest that the p120-catenin, another catenin-binding partner, might regulate this interaction. It is the first of the catenins to bind to cadherin^66^, and its knockdown completely eliminates cell adhesion^67^. Most cadherins are bound to p120: some $82.6 \pm 3.6 \%$ of VE-cadherin and $75.5 \pm 7.7 \%$ of N-cadherins^68^. X-ray crystallography shows that it likely forms oligomers with filaments organised in a crystallographic screw, with a pitch of 17.2nm and 3 residues per turn^69^. This naturally would allow cadherin-catenin complex interactions to be stabilised with a spacing of 17.2nm. Allowing for sub-optimal packing within a punctate adhesion and the fluorescent labels on the cadherins making the adhesions appear slightly larger, this separation between neighbouring cadherins is consistent with punctate adhesions with 6 cadherins and $\approx 50$nm wide as seen by Wu et al.^24^. The strength of cadherin tail-p120 bonds can be very accurately found to be $J = 11 k_B T$ from the detailed isothermal titration calorimetry measurements conducted by Ishiyama et al.^69^ (see their Figure 6A). This value is stronger than the $J\le 7 k_B T$ suggested for *cis* bond^24^, suggesting that p120-cadherin bonds form preferentially prior to cadherin-cadherin *cis* bonds. (Note that there is a possibility that the oligomerization of p120 might have been a crystallisation artifact, as discussed in^69^. However, because recent work by? supports a role of p120 in E-cadherin clustering, we consider the geometry described in this paragraph to be the best model for nearest-neighbour cadherin-cadherin interactions in the remainder of this article.).

### Distance between cadherins and constraints for cadherin clustering

In the *in-vitro* experiments of cadherin clustering by Thompson et al.^19^, the density of cadherin is quite low at ca. $1000 \mu \text {m}^{-2}$. Moreover, because the spacing between cadherins is also much lower at ca. 7 nm, the occupation of a lattice gas where this is the spacing between sites would be quite low. They find that aggregation occurs above a critical concentration of cadherin. This will be the configuration for our Model I investigated below.

The reported density of cadherins within cell edges is much higher at ca. $3500-85000 \mu \text {m}^{-2}$ (range of average to maximal densities)^24^. If the cadherins are uniformly distributed at the developing edge prior to their aggregation, they would be spaced on average ca. 18mm apart. In a lattice gas model of cadherin where the distance between neighbouring cadherins is set by the size of p120 molecule, this would correspond to a fully occupied lattice with every site filled by a cadherin. Here, an increase in the area of the cell edge might lower the average concentration of cadherins and lead to the formation of the first punctate adhesions due to nucleated adhesions becoming more thermodynamically favoured than a uniform distribution of cadherins. This paradigm (which we call the Model II below) resembles the nucleation of integrins on the leading edge of a spreading cell^26^, but here it is fundamentally a 2D process.

To simplify terminology and to make the comparison with the aggregation of integrin complexes more apparent, we will henceforth use the term “adhesion complex” to denote a cadherin-$\beta$-catenin complex, initially not necessarily bound to the cytoskeleton within the cell (Fig. 1).

We seek to examine the initial nucleation of cadherin clusters revealed in super-resolution microscopy, and why there appear to be two distinct populations of larger adhesive clusters as well as smaller non-adhesive clusters^24^. This occurs before the aggregation coarsens into large adherens junctions^70^.

## The models and methods

We recently showed^26^ how attractively coupled integrin adhesion complexes modelled on a 1D lattice undergo a density-dependent phase transition if their initially high concentration decreases past a critical value as the lattice length increases while their number is held constant. This was to model the development of nascent focal adhesions during cell spreading and correctly predicted the number of adhesion clusters.

**Figure 2:**
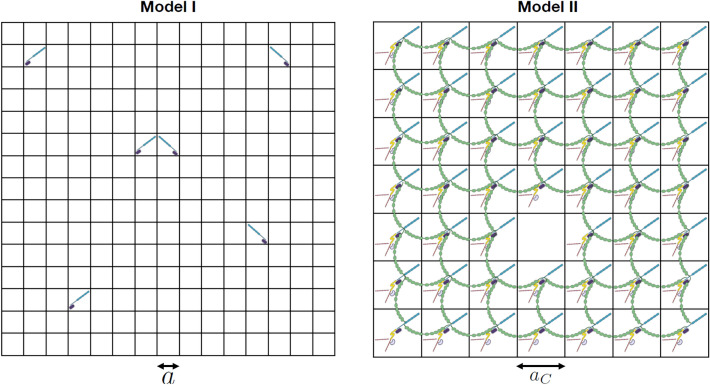
Left panel, **Model I**: Low concentration of cadherins, interacting via *cis* bonds, separated by a lattice spacing $a=7$ nm, consistent with the scenario considered by Thompson et al. in their experiments^19,55^. In this figure, only the two cadherins in neighbouring cells have an interaction energy *J* determined by the *cis* bond strength ca. $5 k_B T$. Clusters become more favourable than a random distribution when the concentration increases past a critical concentration (gas-liquid phase transition). Right panel, **Model II**: High concentration of cadherins, with interactions between neighbouring complexes dictated by cytoplasmic proteins, possibly p120 (long green molecule), see discussion above. p120 increases the spacing between cadherins to $a_C =17.2$ nm^69^. In this figure, only the cadherins around the empty lattice site have less than the maximal interaction energy with neighbouring sites. In this model, the phase transition occurs when the cadherin concentration decreases past a critical value.

The analogy between integrin and cadherin bonding suggests that a lattice gas model might be applicable to the nucleation of cadherin clusters. However, the cell-cell interface is fundamentally 2D (as opposed to integrins concentrated on the rim of the contact area). As commented above, the varying densities of cadherin clusters, as well as the presence or lack of cytoplasmic linker molecules such as p120 to bind neighbouring cadherin molecules more strongly than the *cis* interactions, means that there are at least two different mechanisms at play for the aggregation of cadherin. This leads us to consider two different situations, see Fig. 2. First (Model I), cadherin monomers or already formed small punctate adhesions (for the purposes of the model, adhesion complexes) can aggregate above a critical density as their initially low concentration increases (in line with a gas-liquid condensation and different from the integrin transition). Second (Model II), cadherin complexes can aggregate below a critical density as their initially very high concentration decreases (similar to the situation for integrin^26^).

All of these models have a common core element. We model the contact plane between two cells as a 2D lattice with Neumann boundary conditions, with a total number of lattice sites *A*, and total area $\Sigma =a^2A$, with *a* the lattice spacing or the distance between cadherin complexes. Changes in the density of cadherins are introduced by the adiabatic change in the area of the lattice. The Hamiltonian of two attractively coupled cadherin complexes within one cell is an adaptation of the Ising model:

1 $$\begin{aligned} H = - J \Sigma _{\langle ij \rangle } \eta _i \eta _j \ , \end{aligned}$$

where the sum is over nearest-neighbor pairs and *J* is the bond strength between neighbouring cadherins, provided either by cadherin *cis* bonds or by their pairwise interaction with p120. The variable $\eta$ keeps track of the occupation number of each site:

2 $$\begin{aligned} \eta _i = {\left\{ \begin{array}{ll} &{} 1 \ \ \ \ {\text {adhesion complex present} } \\ &{} 0 \ \ \ \ {\text {adhesion complex absent}} \end{array}\right. } \ \ \ \ . \end{aligned}$$

We show in the Supplementary Materials that a cytoskeletal pulling force, contributing to the Hamiltonian with a linear term $- \Sigma _i h \eta _i$, does not effect the size or distance between clusters, or the concentration at which aggregation begins.

In order to examine the spatial clustering of cadherins, we need to derive the Ginzburg-Landau action of the density distribution of cadherin units. In addition, the diffusion time over the area of membrane that separates the punctate adhesions (50–100 nm) is ca.1 s, similar to the growth time of the cluster (see below). The onset of aggregation is faster still, so the number of cadherins within the area which collapses into a single adhesion (the local *N*/*A* value) should not change substantially at this crucial point.

Over a sufficiently short time interval, the contact area can be assumed constant, and we need not worry about a changing expression for the partition function. The single-molecule partition function for the lattice gas model with Hamiltonian (1) is:

3 $$\begin{aligned} Z_i = \Sigma _{\eta _i = \{0,1\}} e^{-\beta \left[ - J \Sigma _{\langle j \rangle } \eta _j \right] \cdot \eta _i} \ , \end{aligned}$$

where the sum runs over all of the *A* sites in the contact area. The full partition function is the product of all $Z_i$, subject to the constraint of the constant total number of individual cadherin units, $N = \Sigma _i \eta _i$. The Supplementary Part B gives the calculation of this partition function using the auxilliary fields method. There, we introduce a site-specific variable $\rho _i$, whose expectation value is the average occupation of a site $\langle \eta _i \rangle$, and later transform this into a continuous density $\rho (s)$ which depends on a the position *s* in the contact area.

The calculation of $Z_{\text {tot}} = \delta \left( \Sigma _i \eta _i - N\right) \Pi _{i=1}^{A} Z_i$ is exact, but rather unwieldy. We need to make two strong assumptions (see the end of Supplementary Part B) if we want a manageable form for the effective action $S[\rho ]$. First, our two models require different assumptions regarding the concentration of sensors. In Model I, the concentration of sensors is assumed low, so that the probability of a single site being in the ‘empty’ state is much greater than that of being in the ‘filled’ state. Conversely, in Model II, we require that the concentration of sensors be very close to maximal. Second (Supplementary Parts C and D for the treatment of Models I and II, respectively), we look at the nucleation of clusters, where the non-uniformity amplitude is small, so we can work with the series expansion of *S* in terms of density fluctuations $\phi = \rho -N/A$. Note that first-order terms in the new variable $\phi$ average to zero, and only result in a constant shift in the action. This is why the strength of the cytoskeletal pulling force encapsulated in the field term *h* does not change the kinetics of cadherin punctate adhesion aggregation.

In Supplementary Part E, we obtain the action $S[\rho _i]$, transform it into Fourier space, make it continuous, and finally transform back into real space (the last operation generating the spatial gradients). It has recognizable features of the Ginzburg-Landau theory, where we retain cubic and quartic terms in the order parameter expansion:

4 $$\begin{aligned} S[\mathbf {\phi }] = \int d \mathbf {s} \left[ \frac{r_0}{2} \phi ^2(\mathbf {s}) + \frac{c_0}{2} \left[ \nabla \phi (\mathbf {s})\right] ^2 + \frac{t_1}{3!}\phi ^3(\mathbf {s}) + \frac{u_1}{4!}\phi ^4(\mathbf {s}) + \frac{u_2}{4!} \phi ^2(\mathbf {s}) \left( \int d \mathbf {s}' \phi ^2(\mathbf {s}') \right) \right] \ , \end{aligned}$$

where all lengths are scaled by the size of the individual sensor *a*, and the coefficients are listed in Supplementary Part E. Specifically, the two quadratic-order coefficients take the forms, Eqn. (E.9):

5 $$\begin{aligned} r_{0,\iota } = 4 \beta J \left( 1 - 4 g_{2,\iota } \beta J\right) \ ; \ \ \ c_{0,\iota } = 8 g_{2,\iota } \beta ^2 J^2 - \beta J \ , \end{aligned}$$

where $$g_{2,I}= {N}/{A} - {N^2}/{(A+N)^2} $$ in the Model I from Eqn. (C.5), and $$g_{2,II}= {A(3A-2N)}/{(N-2A)^2} - {N}/{A} $$, in the Model II from Eqn. (D.5). The control parameter (replacing the temperature in the classical theory of phase transitions) is the ratio *N*/*A*, whose value can change as the membrane area quickly adapts, *e.g.* during cytokinesis. The gradient coefficient $$c_0$$ remains positive, but the main ‘control’ coefficient $$r_0$$ could become negative at a critical value of *N*/*A* (where $$g_2=k_BT/4J$$) and cause the cadherin distribution to become unstable. Note that near this transition point $$c_0$$ takes the value $$c_0 = \beta J$$.

## Results and comparison with experimental data

In our model, the aggregation transition occurs when $r_0(J, N/ A) = 0$. In Model I (low cadherin concentration) applied to the nucleation of *in vitro* clusters of cadherins spaced some 7 nm apart with an interaction energy of $J = 5 k_B T$, this gives $N/A \approx 0.05$ in a 2D lattice model of cadherin aggregation. This is our first key result, and it matches well with the *in-vitro* observation that cadherin clusters form when their surface density increases past $1100 \ \text {cadherins} \ \mu \text {m}^{-2}$^55^ (this is different from the fraction ca.0.01 of the maximum cadherin surface density which they report, because they consider a much tighter packing of cadherins to within 3 nm of their neighbours). While we will try to estimate the number and size of the adhesions below, we will see that it is difficult here as it is not clear how fast *N*/*A* changes for this particular experimental setup.

**Figure 3:**
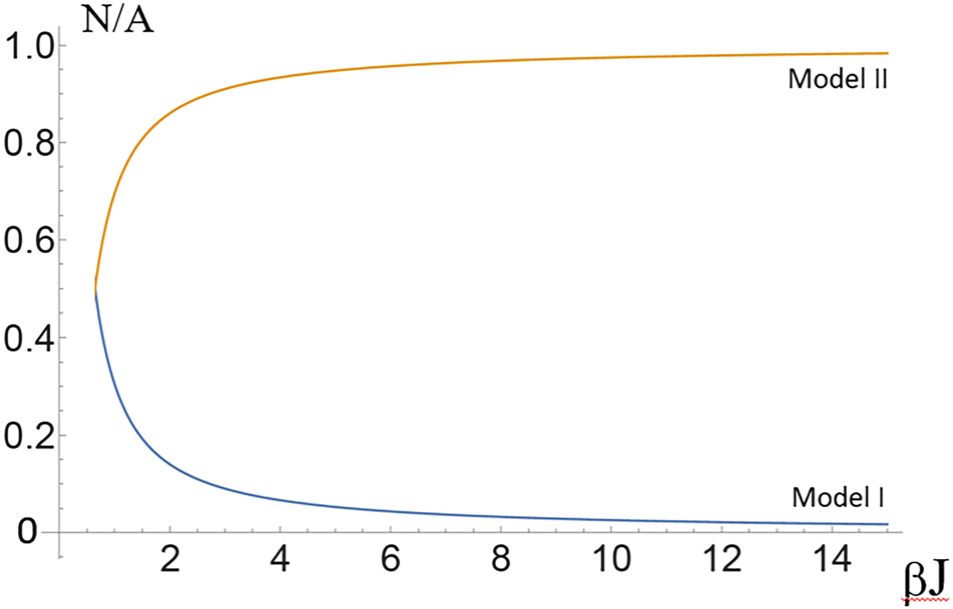
Plot of the lattice site occupation at which clustering begins as a function of the interaction energy *J*. When the concentration of cadherin is low (Model I), a uniform distribution of cadherin complexes can become unstable as the area of the membrane decreases and results in an increase in the concentration of cadherin. The phase transition that occurs in Model II (high cadherin concentration) is symmetrical to this first about $N/A = 0.5$; as the concentration of cadherin decreases, by increasing the area of the membrane, clusters begin to form.

In Model II (high cadherin concentration), used to analyse the nucleation of cadherin clusters interacting via p120 *in vivo* during cytokinesis, $J = 11.0 k_B T$ and the spacing between cadherins is now 17.2 nm, the length of the helical repeat of p120. Here, we find, using the formula for $g_{2,II}$ above, that the transition occurs when $N/A \approx 0.977$, or very close to a uniform concentration of ca. 3400 molecules $\mu \text {m}^{-2}$, which happens to be very close to the average cell-edge density reported by Wu et al. 2015. In this mechanism, cadherin complex nucleation would occur on contact surfaces as soon as the surface becomes large enough that the density of cadherin drops below a certain value. This is incidentally a good mechanism for cells to strengthen large contact areas. The transition lines (the solutions of $r_0=0$) for both models are plotted in Fig. 3.

### Spatial frequency of fluctuations

Near the transition point, the Ginzburg-Landau action Eq. (4) can be approximated by its quadratic terms. The time-dependence of the concentration fluctuation near the transition point can be described by the Cahn-Hilliard equation^71^:

6 $$\begin{aligned} \frac{\partial \phi }{\partial t} = D \nabla ^2 \left( r_0 \phi - c_0 \nabla ^2 \phi \right) \ \ , \end{aligned}$$

where *D* is the (not yet dimensional) cadherin diffusion coefficient in the plane. We impose Neumann boundary conditions on a 2D rectangular cell interface (a reasonable approximation for the lateral surfaces of epithelial cells for instance), which makes the time- and the spatial coordinates fully separable, and gives the Cahn-Hilliard solution:

7 $$\begin{aligned} \phi (x,y,t) = \frac{1}{2}A_0(t) + \Sigma _{\mathbf {k}} A_{\mathbf {k}} (0) e^{-D \frac{\mathbf {k}^2}{4} \left( r_0+\frac{c_0 \mathbf {k}^2}{4} \right) t} \cos {\left( \frac{k_x x}{2} \right) }\cos {\left( \frac{k_y y}{2} \right) } , \end{aligned}$$

where $\mathbf {k} = (k_x, k_y)$ is the wavevector, directly related to the numbers $(m_x,m_y)$ of peaks along the two spatial directions in our contact plane: $\mathbf {k} = (2 \pi m_x/L_x,2 \pi m_y/L_y)$, where the sizes of the lattice in the x and y directions are $L_x$ and $L_y$ respectively, with $L_x L_y = A$. The size of the fastest growing wavevector, which corresponds to the oscillation length scale that maximizes the exponential term above, is

8 $$\begin{aligned} |\mathbf {k} |_{\text {max}} = \sqrt{{-2 r_0}/{c_0 }}\ . \end{aligned}$$

A range of mode numbers $(m_x,m_y)$ satisfy the conditions above. Assuming, as experiments suggest in Fig. 1, that the punctate adherens junctions are roughly equidistant from each other in both directions, we find that the wavenumber of the fastest-growing mode satisfies:

9 $$\begin{aligned}{}&k_{x,\text {max}} \approx k_{y,\text {max}} \Rightarrow \frac{m_{x,y}}{L_{x,y}} = \frac{1}{2 \pi } \sqrt{-\frac{r_0}{c_0}} . \end{aligned}$$

The total number of clusters within the patch of cell-cell contact area is the product $M = m_x m_y$.

### Mode destabilisation time

A density fluctuation mode cannot grow until it becomes unstable, that is, when the second-order terms in the Ginzburg-Landau action become negative for that value of *k*. Lower *k* wavevectors become unstable closer to the density at the transition point ($r_0=0$), while at higher *k* wavevectors the increasing interface energy requires a larger negative $r_0$ to destabilize the homogeneous density. Alternatively, large diffuse clusters form before smaller clusters. Assuming a steady rate change of the contact area, we find in Supplementary Part F that the largest number of clusters *M* in the lattice, for which the combined second-order Ginzburg-Landau term is negative, is proportional to the time $t_1$ that has elapsed since the cadherin density crossed the transition point $r_0 = 0$. Reintroducing dimensional length scales, we find this linear relation:

10 $$\begin{aligned} M \approx \beta J \Psi _{\iota }(\beta J) \frac{t_1 | \dot{\Sigma }|}{a^2} \ , \end{aligned}$$

where $\dot{\Sigma }$ is the rate of decrease in the cell-cell contact area, and $\Psi _{\iota }(\beta J)$ is a complicated function obtained from the series expansion with a different form for each model $\iota =$I or II, Eqns. (G.2,H.2).

### Mode growth time

For the fastest growing mode, $r_0 = -c_0 k_{\text {max}}^2/2$, Eq. (8), so we find that the mode has a characteristic exponential growth time $t_2$ which dictates how fast the Ginzburg-Landau free energy is minimized. We find this time by substituting into the exponent in the Cahn-Hilliard solution (7):

11 $$\begin{aligned} e^{-D \frac{k^2}{4} \Big (r_0 + \frac{c_0 k^2}{4} \Big ) t}\Big |_{\text {max}} = e^{D \frac{k^4 c_0}{16} t} \end{aligned}$$

Substituting the constants $r_0$ and $c_0$, and recovering the proper dimensional length scales via the spacing *a*, we find that the growth time of the mode becomes:

12 $$\begin{aligned} t_2=\alpha \frac{A^2 a^2}{4\pi ^4 M^2 D \beta J} \ , \end{aligned}$$

i.e. inversely proportional to $M^2$, with $\alpha$ a proportionality constant of order 1.

### Density of punctate adherens junctions

The total time for a mode to first become unstable and then reach the minimum in the Ginzburg-Landau free energy landscape is of the order:

13 $$\begin{aligned} t_{\text {tot}} = t_1 + t_2 = K_1 M + K_2 M^{-2} \end{aligned}$$

where $K_1$ and $K_2$ are the proportionality constants in Eqs. (10,12). The total time is minimized when the number of clusters across the contact area is:

14 $$\begin{aligned} \frac{d t_{\text {tot}}}{d M}&= K_1 - 2 K_2 M^{-3} = 0 \Rightarrow M^* = \left( \frac{2 K_2}{K_1} \right) ^{1/3} . \end{aligned}$$

We make the proportionality constants explicit and find that the number of adherens junctions per unit area of cell-cell interface is given by

15 $$\begin{aligned} n_{\text {s}} = \frac{M^*}{a^2 A} = \left( \alpha \frac{\Psi _{\iota }(\beta J)}{2 \pi ^4 a^4 D} \frac{|\dot{\Sigma }|}{\Sigma }\right) ^{1/3} \end{aligned}$$

This is the second main prediction of this paper. We find in the Supplementary that $\Psi _{\iota }(\beta J)$ is an increasing function of *J* at low interaction energy, before plateauing at high interaction energy. This means that the number of adherens junctions primarily depends on the lattice spacing *a*, and to a lesser extent the diffusion constant *D*, and the fractional rate of change of the contact area $|\dot{\Sigma }|/\Sigma$.

**Figure 4:**
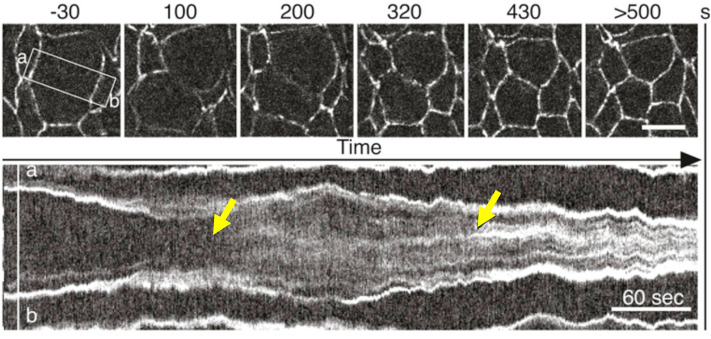
Evolution of cadherin density on the apical edge of a dividing epithelial cell during cytokinesis, reproduced with permission from^73^. Cadherins are labelled with E-cad::GFP fluorescent markers; the second panel (kymograph) shows the density along the [a-b] cross-section, illustrating how the interface area first expands and then contracts. During this time, the intensity profile of the cadherins increases sharply at two times indicated by arrows. Previous work on integrin and paxillin has shown an increase in the intensity of the fluorescent marker as the time of the first formation of focal adhesions^81^; this increase in intensity could arise due to an increase in the organisation of the molecular structure and different collective fluorescence properties as seen in certain luminescent biomaterials^82^. We suggest that the two sharp increases here arise due to the formation of small punctate adhesions, possibly followed later by the further aggregation of those clusters with transmembrane adhesions into larger clusters^24^. During both the expansive and contractile phases, the length of the apical edge of the new contact area between daughter cells changes at a rate of $(|\dot{\Sigma }|/\Sigma )^{0.5} \approx 20 \% \ \text {min}^{-1}$.

Now, let us apply our two models to make an experimental comparison for the number (and size) of the clusters. One classic example which requires cadherin clusters to form occurs during cytokinesis at the end of cell division^72^. Kymographs of the cross-section of the a dividing epithelial cell^73,74^, see Fig. 4, show that the fractional change in the area of the cell interface is $|\dot{\Sigma }|/\Sigma \approx 20 \%$ per minute, both during an initial expanding phase after the contractile ring dissipates^75^, and as the cell-cell contact area readjusts later.

We suggest that both sharp cadherin intensity changes seen in Fig. 4 correspond to different phases of aggregation. The first occurs just after the dissolution of the contractile mitotic ring^75^, with a decreasing concentration of sensors as the area of the cell expands. This corresponds to a situation where Model II might be applicable. To make an experimental comparison, we use the aggregated cadherin spacing $a_C$ = 17.2 nm^69^, diffusion constant $D_C = 3\cdot 10^{-3} \mu \text {m}^2/$s^76^, density at transition of $N/A = 0.977$ (see above), $J_C=11 k_B T$, and proportionality constant $\alpha = 1$ for the sake of simplicity (see Table 1). The fractional shrinking rate of a patch of the cell-cell contact area $(|\dot{\Sigma }|/\Sigma )$ depends on the cellular process and the type of cell, but here we estimate $|\dot{\Sigma }|/\Sigma \approx 20 \% \ \text {min}^{-1}$. We find here that $n_{\text {s}} \approx 30 \pm 5 \ \text {clusters} \ \mu \text {m}^{-2}$. This is consistent with the median spacing between the larger apical and lateral cadherin clusters observed by Wu et al. in their Fig. 2^24^.

Wu et al.^24^ reported a very large difference in density between these larger clusters and punctate adhesions observed elsewhere in the cell. Indeed, although the mean density of cadherins was 3500 cadherins $\mu \text {m}^{-2}$, the median density of cadherins per cluster was only 6. This indicates the presence of a large population of small non-adhesive clusters that could have formed via a separate mechanism. As we see in Eq. (15), the predictions for the density of clusters in both of our Models for low and high cadherin concentration are similar, except for the function $\Psi _{\iota }(\beta J)$, which we find in the Supplementary Materials is approximately constant at high $J>10 k_B T$, and that it is similar for the cadherin *cis* bond or the cadherin-p120 bond. In fact, as the new cell-cell interface expands during the initial phase of cytokinesis, we expect the rest of the cell membrane to contract, and for a possibly small concentration of cadherins to locally increase, at perhaps a slightly slower rate than the $|\dot{\Sigma }|/\Sigma \approx 20 \% \ \text {min}^{-1}$ seen at the new cell-cell edge. If *cis* bonds (strength $J = 5 k_B T$) control these interactions (perhaps because all of the p120 molecules are co-localised with the larger cadherin clusters in the denser regions), the lattice spacing would be smaller at $a \approx 5-7$nm^52,55,59,77^, and so the corresponding density of clusters would be higher at $n_s \approx 60-100$, . The minimum cadherin density at which these small clusters would form is when $r_0(\beta J) = 0$, or $N/A \approx 0.05$. These clusters would be much smaller, perhaps with only 10-15 cadherins per cluster. This is close to the densities and number of cadherins per small non-adhesive punctate cadherin cluster reported by Wu et al. in their Fig. 4^24^.

**Table 1:** Summary of the physiological values used in the model.

Parameter	Name	Value	Uncertainty	References
Cadherin lattice spacing	*a*	7 nm	± 1 nm	^52,55,59,77^
Cadherin-catenin unit lattice spacing	$a_C$	17.2 nm	negligible	^69^
Purified cadherin diffusion coefficient (or catenin-minus)	D	$1.1\cdot 10^{-2} \mu \text {m}^2/$s	$^{+4}_{-0.7}\cdot 10^{-2} \mu \text {m}^2/$s	^78^
Cadherin diffusion coefficient (macroscopic)	$D_C$	$2.6\cdot 10^{-3} \mu \text {m}^2/$s	$\pm 1.1\cdot 10^{-3} \mu \text {m}^2/$s	^76^
Cadherin diffusion coefficient (microscopic)	$D_C$	$3.3\cdot 10^{-3} \mu \text {m}^2/$s	$\pm 2.9\cdot 10^{-3} \mu \text {m}^2/$s	^76^
Cytoskeleton-bound coefficient (or fusion)	$D_{CC}$	$0.3\cdot 10^{-3} \mu \text {m}^2/$s	$^{+1}_{-0.2}\cdot 10^{-3} \mu \text {m}^2/$s	^78^
Cis interaction energy	J	$$ 5 k_B T$$	$$\pm 2~k_B T$$	^59,63^
Cadherin-p120 interaction energy	$$J_C$$	$$ 11.0~k_B T$$	negligible	^69^
Fractional change of cell-cell contact area	$|\dot{\Sigma }|/\Sigma$	$\approx 20 \% \text {min}^{-1}$	$\pm 5 \% \text {min}^{-1}$	Figure 2^73^
Density at the phase transition, Model I	N/A	0.05	$\pm 0.01$	(see above and^55^)
Density at the phase transition, Model II	N/A	0.977	$\pm 0.01$	(see above and^24^)
Arbitrary exponential growth parameter	$\alpha$	1–10	$*$	NB$\dagger$

Note the large standard deviation in the cadherin diffusion constants. This arises due to the different modes of cadherin movement^76^; however, the uncertainty on the average value is much less than this standard deviation would suggest. We use the data from Figure 11 from Sako et al.^78^ to estimate the asymmetrical uncertainties on *D* and $D_{CC}$, and report the median value which seems more meaningful. For the diffusion rate of a cluster of 6 cadherins, we estimate the degree of reduction in *D* to be slightly less than an order of magnitude^79^. $\dagger$ NB: Depends on the tolerance on how far the coarsening has progressed. Here we choose a factor of *e* (ca. $63\%$ growth), whereas simulations with a $1\%$ tolerance^80^ give a value of $\alpha$ closer to 10. It seems clear that cadherin clustering should become visible before coarsening completes.

Another intriguing possibility is that the huge variation in the cadherin diffusion constant *D* reported by^76,78^ (and see Table 1) might help set up separate distributions of cadherin clusters. In their Figure 11, Sako et al.^78^ find that the diffusion constant of wild-type cadherins can vary by up to three orders of magnitude. If all other parameters remain the same, it is quite possible that a ‘slow’ cadherin population would form with 10 times more clusters ($n_s$ 10 times higher in Eq. 15). Given that some of this variability in *D* disappear in the absence of links to the cytoskeleton^78^, it would be interesting to see if either population of smaller non-adhesive or larger adhesive punctate junctions reported by Wu et al.^24^ disappears without cytoskeletal links. Indeed, there appears to be a role in hybrid live cell-supported membrane systems for actin-rich filopodia contributing to a decrease in cadherin mobility and thereby helping to develop adherens junctions^83^, so we expect super-resolution microscopy of such a system to help explain the origins of these two populations of punctate adhesions.

Even though there might be some uncertainty in the values of physiological constants used to evaluate Eq. (15), the cube-root dependence of the number density on these constants makes a large error unlikely: any one parameter value would need to be off by a factor of 1000 for there to be 10 times fewer or more punctate adhesions. Note that we can also use this figure to estimate the total growth and destabilisation time to be of the order of 5 seconds, once the cadherin distribution becomes unstable. This would account for the quite sharp transition to a higher cadherin instensity in the kymograph at the time indicated by the arrow.

## Conclusions

In this paper, we built a 2D model for the aggregation of adhesion units with a short-distance attractive interaction, which is applicable to all cell contact areas if the problem is reduced down to a sufficiently small and uniform patch (locally, with zero curvature and applied force variation), so we expect our results to be more universally valid. The advantage of treating cadherin adhesion complexes as a specific example is that there is a large body of experimental and computational work from which to test our results. Our predictions for the transition density of cadherins above which clusters could form, as well as for the number density of cadherin clusters, were independent of each other. Together, they strongly suggest that cadherin cluster nucleation initially occurs via a density-dependent phase transition.

Previous studies have analytically explained how cadherin *trans* bonds can help set up large scale adherens junctions^23^, and new work has computationally shown that cadherin cis-bonds help develop punctate adherens junctions^63^. In this work. we have laid out for the first time an *analytical* explanation for how *punctate* cadherin adherens junctions can form.

While we looked at the case of cadherin nucleation, more generally, any distribution of attractively-coupled cell membrane molecules in the low concentration limit can undergo a gas-liquid (condensation) phase transition in the form of density-dependent aggregation. Because of this, we could apply this method to the more general problem of the formation of membrane-bound organelles.

## Supplementary Information

Supplementary Information 1.

## Data Availability

All data generated or analysed during this study are included in this published article [and its supplementary information files].
