# Supplementary material for: Nucleation of cadherin clusters on cell-cell interfaces
Source: Sci Rep. 2022 Nov 2;12:18485. doi: 10.1038/s41598-022-23220-x (PMC9630535; doi:10.1038/s41598-022-23220-x)
Supplement: Supplementary file 1 — Supplementary Information 1. [file 41598_2022_23220_MOESM1_ESM.pdf]

Nucleation of cadherin clusters on cell-cell interfaces
SUPPLEMENTARY INFORMATION

A. CALCULATIONS FOR MEMBRANE FLUCTUATION MODEL VS DIRECT BONDING MODEL

Fenz et al.[1] quantify the binding and unbinding rates of cadherin monomers to their trans binding partners in their Supplementary materials. They adjust expressions from Dembo[2] and Bell[3] to express these rates as depending on the relative distances between the two membranes $h(x_i, t)$:

$$k_{\rm on} = k_0\sqrt{\frac{\lambda_c\alpha^2}{4\pi}}\exp\Big[-\frac{\lambda_c}{4}(\Delta h_0^{x_i}-\alpha)^2\Big] \qquad \text{(A.1)}$$

and

$$k_{\rm off} = k_0\exp\Big[\frac{\lambda_c}{2}\Delta h_0^{x_i}-\frac{\lambda_c\alpha^2}{4}-\epsilon_b\Big] \qquad \text{(A.2)}$$

where k_0 is the intrinsic reaction rate, α the binding range between the outer cadherin domains (taken to be 1nm in their text), ϵ_b the binding enthalpy of the bond, λ_c the elastic constant of the cadherin and $\Delta h^{x_i} \equiv h_0^{x_i} - 2l_0$ the instantaneous separation between the outer cadherin domains.

For a cadherin domain to stop growing indefinitely, local membrane fluctuations need to be sufficiently large that $k_{\rm off} = k_{\rm on}$. This occurs when:

$$\Delta h_0^{x_i} = \Big[\frac{4}{\lambda_c}\Big(\epsilon_b + \frac{1}{2}\ln\frac{\lambda_c\alpha^2}{4\pi}\Big)\Big]^{1/2} \quad . \qquad \text{(A.3)}$$

Here, the two important parameters are the binding enthalpy ϵ_b and the cadherin stiffness λ_c. Fenz et al.[1] give a value $\epsilon_b = 7k_BT$, which is on the lower end of reported stiffnesses (wild-type cadherin bonds strengthen with time from 9 to $13k_BT$[4]). They also use a value of $\lambda_c = 5\times10^{-2}k_BT$ nm^{-2} for cadherin stiffness. This value is on the upper end of stiffness values extrapolated from single molecule cadherin AFM studies[5], which show that force-extension curves are best-fitted by a persistence length $l_p = 0.5\pm0.1$nm and a domain length $L_0 \approx 60$nm. In the linear regime, the WLC model yields a stiffness $\lambda_c \approx \frac{k_BT}{l_pL_0} \approx 3\times10^{-2}k_BT\text{nm}^{-2}$ for cadherin.

Substituting these values into Eqn. A.3 above, we find that the size of the membrane fluctuations required for two cadherin clusters to dissociate is $\Delta h_0^{x_i} \approx 30-40$nm.

This value must now be compared with the observed sizes of membrane fluctuations in cells - which have a well-defined cytoskeleton that modifies the amplitude of membrane fluctuations as opposed to GUVs used in experiments and simulations by Fenz et al.[1]. Biswas et al.[6] find that spatial fluctuations in cells adhering to flat surfaces fluctuate spatially by 7.2 ± 1.5 nm and temporally by 4.9 ± 0.7 nm in adhesive regions (First Branch Regions, which cover most of the cell-surface contact). This value matches with similar results reported by Dos Santos et al.[7], who also find in their Figures 5 and 6 that fluctuations are lower in focal adhesions - regions where the membrane is closer to the surface.

Cadherins adhere cells to their neighbours, rather than to a smooth matrix, so local fluctuations in the inter-membrane distance will result from active cortex-mediated fluctuations from both cells. Whilst it is very difficult to exactly quantify this effect, we expect that it should increase the amplitude of local fluctuations in the separation between the two membranes (assuming two independent Gaussian distributions of fluctuations) by $\approx\sqrt{2}$, leading to local $10-15$ nm fluctuations in the inter-membrane separation within adhesion regions. This is much less than the $30-40$ nm figure required for the formation of cadherin *trans*-bonds. In other words, most cadherin trans bonds are quickly formed within the larger adhesion area, and thousands of cadherins are brought together by diffusion until a sharp boundary forms around the entire zonula adherens.

However, within the large adhesion region, most cadherin *trans* dimers have now formed, and simulations by Fenz et al.[1] show that sufficiently small height differences (<25 nm) between cadherins do not lead to any structure formation, regardless of the size of membrane fluctuations (see Figure SI 4 in their text). Because the membrane fluctuations, and hence the height differences between cadherins are much smaller than 25 nm in larger adhesive areas, cadherins within these regions do not form dense micro-structures without another mechanism, namely direct *cis* bonds. These computational predictions are corroborated by experiments which show that punctate adhesions do not develop when *cis*-abolishing V81D/V175D mutations are introduced.

B. CALCULATION OF THE PARTITION FUNCTION

The full partition function is the product of A partition functions for each site, subject to the constraint $N = \Sigma_i\eta_i$ of the constant total number of individual sensors. This condition can be expressed with a delta function, and rearranged into matrix form as:

$$\begin{aligned} Z_{\rm tot} &= \delta(\Sigma_i n_i - N)\Pi_{i=1}^A Z_i \qquad \text{(B.1)}\\ &= \frac{1}{2\pi}\int_{-\infty}^{+\infty} d\tilde{\mu}\,\Big(e^{-i\tilde{\mu}N}\Sigma_{\{\eta_i=\{0,1\}\}}e^{(\frac{1}{2}\eta^T\tilde{\mathbf{J}}\mathbf{n}+i\tilde{\mu}^T\eta+h^T\eta)}\Big), \end{aligned}$$

with the non-dimensional coupling constant $\tilde{\mathbf{J}} = \beta\mathbf{J}$. Using the N-dimensional Gaussian integral identity, we can write the second exponential term in Eqn.(B.1) as:

$$e^{\frac{1}{2}\eta^T\tilde{\mathbf{J}}\eta} = \frac{\int D[x]e^{-\frac{1}{2}\mathbf{x}^T\tilde{\mathbf{J}}^{-1}\mathbf{x}}e^{\pm\mathbf{x}^T\eta}}{\int D[x]e^{-\frac{1}{2}\mathbf{x}^T\tilde{\mathbf{J}}^{-1}\mathbf{x}}}, \qquad \text{(B.2)}$$

where $\mathbf{x}$ is the Hubbard-Stratonovich vector field, which we need to later obtain the order parameter description. Note the ambiguity in the sign of the term with the exponent linear in $\mathbf{x}$ in Eqn. (B.2); both formulations will later be shown to be identical when expressed with respect to the physical mechanosensor density.

Treating $\mathbf{x}$ as independent of η, we can factorise the partition function (B.1) as follows:

$$Z = \frac{\int D[x] e^{-\frac{1}{2}\mathbf{x}^T \tilde{\mathbf{J}}^{-1}\mathbf{x}} \frac{1}{2\pi}\int_{-\infty}^{+\infty} d\tilde{\mu}\ e^{-i\tilde{\mu}N} \Sigma_{\{\eta_i\}} e^{(i\tilde{\mu}^T \pm \mathbf{x}^T + h^T)\eta}}{\int D[x] e^{-\frac{1}{2}\mathbf{x}^T \tilde{\mathbf{J}}^{-1}\mathbf{x}}} \tag{B.3}$$

The integral of the linear part of Eqn.(B.3) can be simplified:

$$\begin{aligned} &\frac{1}{2\pi}\int_{-\infty}^{+\infty} d\tilde{\mu}\ e^{-i\tilde{\mu}N} \Pi_{i=1}^{A} \Big[\Sigma_{\eta_i} e^{(i\tilde{\mu}^T + h^T \pm \mathbf{x}^T)\eta}\Big] \\ &= \frac{1}{2\pi}\int_{-\infty}^{+\infty} d\tilde{\mu}\ e^{-i\tilde{\mu}N} \Pi_{i=1}^{A} \Big[\Big(e^{(i\tilde{\mu} + h^T \pm x_i)} + 1\Big)\Big] \ . \end{aligned} \tag{B.4}$$

An action functional $\tilde{S}_{\pm}[\mathbf{x}, \tilde{\mu}]$ can now be defined following the steps of Landau-Ginzburg-Wilson theory:

$$Z = \frac{1}{\sqrt{\det \tilde{\mathbf{J}}}} \int D[x] \int_{-\infty}^{+\infty} d\tilde{\mu}\ e^{-\tilde{S}_{\pm}[\mathbf{x},\tilde{\mu}]} \ , \tag{B.5}$$

and found to be in the most general form:

$$\tilde{S}_{\pm}[\mathbf{x}, \tilde{\mu}] = i\tilde{\mu}N + \frac{1}{2}\mathbf{x}^T \tilde{\mathbf{J}}^{-1}\mathbf{x} - \Sigma_{i=1}^{A} \ln\big[1 + e^{(i\tilde{\mu} + h^T \pm x_i)}\big] \ . \tag{B.6}$$

The next step is to calculate the expectation value of the Hubbard-Stratonovich field:

$$\langle \pm x_i \rangle_{\tilde{S}} \equiv \frac{\frac{1}{2\pi}\int D[x] \int_{-\infty}^{+\infty} d\tilde{\mu}\ e^{-\tilde{S}_{\pm}[\mathbf{x},\tilde{\mu}]}(\pm x_i)}{\frac{1}{2\pi}\int D[x] \int_{-\infty}^{+\infty} d\tilde{\mu}\ e^{-\tilde{S}_{\pm}[\mathbf{x},\tilde{\mu}]}} \ . \tag{B.7}$$

After introducing an auxiliary N-component column vector $\mathbf{y} = (y_1, ..., y_N)^T$, we can re-express the Hubbard-Stratonovich field components as:

$$\pm x_i = \lim_{\mathbf{y}\to 0} \frac{\partial}{\partial y_i} e^{\pm \mathbf{x}^T \mathbf{y}} \ . \tag{B.8}$$

Then performing Gaussian integration in Eqn. (B.7) modified with $\eta \to \eta + y$, we obtain $\langle x_i \rangle_{\tilde{S}} = \langle [\tilde{\mathbf{J}}' \eta]_i \rangle$ if the sign of the exponent of the linear term $e^{\mathbf{x}^T \eta}$ in Eqn. (B.2) is chosen to be positive; or $\langle -x_i \rangle_{\tilde{S}} = \langle [\tilde{\mathbf{J}}' \eta]_i \rangle$ if this exponent is instead negative.

We need a variable whose expectation value can be identified with the average occupation of a site, which is the order parameter:

$$\rho = \pm \tilde{\mathbf{J}}'^{-1} \mathbf{x} \ , \quad \text{so that} \ \langle \rho_i \rangle_{\tilde{S}} = \langle \eta_i \rangle \tag{B.9}$$

This identifies the discrete field ρ_i as the mechanosensor concentration at site i. We will later transform this to a continuous density ρ which depends on a the position along the adhesion contact ring.

An immediate consequence of defining the density field ρ in Eqn. (B.9) is that the previously noted sign ambiguity is lifted: both formulations lead to identical expressions when expressed in terms of ρ. We may therefore without loss of generality only consider the case where the exponent of the linear term $e^{\mathbf{x}^T \eta}$ in (B.2) is positive.

It is convenient at this stage to define the fluctuation of the Hubbard-Stratonovich field over all A lattice sites: $x'_i = x_i - \frac{1}{A}\Sigma_i x_i$. The corresponding fluctuation of the concentration field about its average value $\rho_a = N/A$ is directly related: $\phi = \rho - \rho_a = \tilde{\mathbf{J}}'^{-1} \mathbf{x}'$.

At equilibrium, the action is maximum with respect to $\tilde{\mu}$, or equivalently with respect to the modified variable $m = i\tilde{\mu}$ (according to the condition of stationary phase). Carrying out the μ-differentiation of Eqn. (B.6) allows us to express m in terms of the Hubbard-Stratonovich field $\mathbf{x}$ and the system variables A and N in equilibrium:

$$\Sigma_{i=1}^{A} \Big(\frac{1}{1 + e^{-(m+h+x_i)}}\Big) = N. \tag{B.10}$$

Model I: Low cadherin concentration

The low-occupancy approximation gives the following condition for the Boltzmann factors:

$$e^{m+h+x_i} \ll 1 \ . \tag{B.11}$$

When $N \ll A$, we can simplify the constraint (B.10) in order to analytically solve it:

$$\begin{aligned} N = \Sigma_{i=1}^{A} \Big(\frac{1}{1 + e^{-(m+h+x_i)}}\Big) &\approx \Sigma_{i=1}^{A} (e^{(m+h+x_i)}) \\ &\approx e^{(m+h+x_a)} \Sigma_{i=1}^{A} e^{x'_i} \quad . \end{aligned}$$

This can be rearranged to:

$$e^{c} = e^{-(m+h+x_a)} = \frac{1}{N}\ \Sigma_{i=1}^{A} e^{x'_i} \gg 1. \tag{B.12}$$

As expected, this is a large quantity if the number of sites is large and the number of sensors is relatively small.

Model II: High cadherin concentration

On the other hand, we find an opposite situation in the high-occupancy approximation:

$$e^{h+m+x_i} \gg 1 \ . \tag{B.13}$$

When $(A - N) \ll A$, we simplify the constraint (B.10):

$$\begin{aligned} N = \Sigma_{i=1}^{A} \Big(\frac{1}{1 + e^{-(h+m+x_i)}}\Big) &\approx \Sigma_{i=1}^{A} (1 - e^{-(h+m+x_i)}) \\ &\approx A - e^{-(h+m+x_a)} \Sigma_{i=1}^{A} e^{-x'_i} \end{aligned}$$

and rearrange it to:

$$e^{(h+m+x_a)} = \frac{1}{A - N}\ \Sigma_{i=1}^{A} e^{-x'_i} \gg 1. \tag{B.14}$$

This is a large quantity if the number of sites and the number of sensors are both large.

C. SERIES EXPANSION OF THE ACTION: MODEL I, LOW CADHERIN CONCENTRATION

By explicitly substituting $m(x'_i)$ as obtained in Eqn. (B.10) into Eqn. (B.6), we can obtain a useful expression for the action $S(x'_i, m)$ in terms of x'_i only. Doing so mathematically incorporates the $N =$ const. constraint on the number of mechanosensing complexes into Ginzburg-Landau action and will allow us to subsequently formulate it in terms of the physical sensor concentration fluctuation ϕ. We begin by writing the exponential $e^{x'_i}$ as a series:

$$e^{x'_i} = 1 + x'_i + \frac{1}{2}x'^2_i + \frac{1}{3!}x'^3_i + \frac{1}{4!}x'^4_i + ... \tag{C.1}$$

Summing over all of the states i gives:

$$e^c = \frac{1}{N}\left(A + \Sigma_i x'_i + \frac{1}{2}\Sigma_i x'^2_i + \frac{1}{3!}\Sigma_i x'^3_i + \frac{1}{4!}\Sigma_i x'^4_i + ...\right)$$

To keep track of the order of fields, we introduce the parameter a through:

$$\begin{aligned}\Sigma_i x'_i &\to a\Sigma_i x'_i \\ \Sigma_i x'^2_i &\to a^2\Sigma_i x'^2_i \\ \Sigma_i x'^3_i &\to a^3\Sigma_i x'^3_i \\ \Sigma_i x'^4_i &\to a^4\Sigma_i x'^4_i \ .\end{aligned}$$

We now expand the above in powers of a:

$$\begin{aligned}&\ln((N)e^c) = \\ &= \ln(A) + \frac{a^2\Sigma_i x'^2_i}{2A} + \frac{a^3\Sigma_i x'^3_i}{6A} + \frac{a^4\left(A\Sigma_i x'^4_i - 3(\Sigma_i x'^2_i)^2\right)}{24A^2} \\ &\Rightarrow m = -x_a - h - \ln\left(\frac{A}{N}\right) - \frac{a^2\Sigma_i x'^2_i}{2A} - \frac{a^3\Sigma_i x'^3_i}{6A} \\ &- \frac{a^4\left(A\Sigma_i x'^4_i - 3(\Sigma_i x'^2_i)^2\right)}{24A^2}\end{aligned} \tag{C.2}$$

A similar trick can be used to track the order of the terms in the expansion of $-\Sigma_{i=1}^A \ln\left[1 + e^{(m+h+x_a+x'_i)}\right]$, substituting in the expression for the Boltzmann factor. We are interested in the expansion of:

$$\begin{aligned}&\Sigma_{i=1}^A \ln\left[1 + e^{(m+h+x_a+x'_i)}\right] = \\ &= A\ln\left(1 + \frac{1}{e^c}\right) + \frac{e^c a^2\Sigma_i x'^2_i}{2\left(e^c+1\right)^2} \\ &+ \frac{e^c a^3\Sigma_i x'^3_i\left(e^c - 1\right)}{6\left(e^c+1\right)^3} + \frac{e^c a^4\Sigma_i x'^4_i\left(1 - 4e^c + e^{2c}\right)}{24\left(e^c+1\right)^4} \ .\end{aligned} \tag{C.3}$$

Substituting in the expansion of the Boltzmann factor e^c, we find the series expansion of this term up to 4th order in a:

$$\begin{aligned}&-\Sigma_{i=1}^A \ln\left[1 + e^{(h+m+x_a+x'_i)}\right] = -A\ln\left(1 + \frac{N}{A}\right) \\ &+ \frac{a^2N^2\Sigma_i x'^2_i}{2(A+N)^2} + \frac{a^3N^2(N+3A)\Sigma_i x'^3_i}{6(A+N)^3} \\ &+ \frac{a^4N^2\left(-3(\Sigma_i x'^2_i)^2((A+N)(5A+N)) + A\Sigma_i x'^4_i\left(7A^2+2AN+N^2\right)\right)}{24A(A+N)^4}\end{aligned} \tag{C.4}$$

Setting the series parameter $a = 1$, we forget for the time being about the explicit dependency of the terms on A and N, and write:

$$\begin{aligned}&mN - \Sigma_{i=1}^A \ln\left[1 + e^{(h+m+x_a+x'_i)}\right] = \\ &= g_{0,I}(A,N,h) - \frac{g_{2,I}(A,N)}{2}\Sigma_i x'^2_i + \frac{g_{3,I}(A,N)}{3!}\Sigma_i x'^3_i \\ &+ \frac{g_{4a,I}(A,N)}{4!}\Sigma_i x'^4_i + \frac{g_{4b,I}(A,N)}{4!}(\Sigma_i x'^2_i)^2 \ .\end{aligned} \tag{C.5}$$

where we make the identifications:

$$\begin{aligned}g_{0,I}(A,N,h) &= -N\ln\left(\frac{A}{N}\right) - A\ln\left(\frac{N}{A} + 1\right) - N(x_a + h) \\ g_{2,I}(A,N) &= \frac{N}{A} - \frac{N^2}{(A+N)^2} \\ g_{3,I}(A,N) &= \frac{N^2(N+3A)}{(A+N)^3} - \frac{N}{A} \\ g_{4a,I}(A,N) &= \frac{N^2(7A^2+2AN+N^2)}{(A+N)^4} - \frac{N}{A} \\ g_{4b,I}(A,N) &= \frac{3N}{A^2} - \frac{3N^2(5A+N)}{A(A+N)^3} \ .\end{aligned}$$

The action (or the effective free energy) can be re-expressed in terms of the average value of the Hubbard-Stratonovich field in order to substitute the above:

$$\begin{aligned}&\tilde{S}_I[\mathbf{x}', \mathbf{x}_a, A, N] = \\ &= \frac{1}{2}(\mathbf{x}_a + \mathbf{x}')^T\tilde{\mathbf{J}}'^{-1}(\mathbf{x}_a + \mathbf{x}') + g_{0,I}(A,N) - \frac{g_{2,I}(A,N)}{2}\Sigma_i x'^2_i \\ &+ \frac{g_{3,I}(A,N)}{3!}\Sigma_i x'^3_i + \frac{g_{4a,I}(A,N)}{4!}\Sigma_i x'^4_i + \frac{g_{4b,I}(A,N)}{4!}(\Sigma_i x'^2_i)^2 \ .\end{aligned} \tag{C.6}$$

We will also need to check that the total contribution of the sixth order term is positive in order for the Hamiltonian to be bounded from below. Using the connection, Eqn. (B.9), between the Hubbard-Stratonovich field x and the density ρ, we finbally obtain the action $S[\rho]$.

D. SERIES EXPANSION OF THE ACTION: MODEL II, HIGH CADHERIN CONCENTRATION

Here, we write the exponential $e^{-x'_i}$ as a series:

$$e^{-x'_i} = 1 - x'_i + \frac{1}{2}x'^2_i - \frac{1}{3!}x'^3_i + \frac{1}{4!}x'^4_i - ... \tag{D.1}$$

Summing over all of the states i gives:

$$e^c = \frac{1}{A-N}\left(A - \Sigma_i x'_i + \frac{1}{2}\Sigma_i x'^2_i - \frac{1}{3!}\Sigma_i x'^3_i + \frac{1}{4!}\Sigma_i x'^4_i - ...\right)$$

Introducing the order parameter a as above

$$\begin{aligned}\Sigma_i x'_i &\to a\Sigma_i x'_i \\ \Sigma_i x'^2_i &\to a^2\Sigma_i x'^2_i \\ \Sigma_i x'^3_i &\to a^3\Sigma_i x'^3_i \\ \Sigma_i x'^4_i &\to a^4\Sigma_i x'^4_i \ ,\end{aligned}$$

we expand the exponential in powers of a:

$$\begin{aligned}&\ln((A-N)e^c) = \\ &= \ln(A) + \frac{a^2\Sigma_i x'^2_i}{2A} - \frac{a^3\Sigma_i x'^3_i}{6A} + \frac{a^4\left(A\Sigma_i x'^4_i - 3(\Sigma_i x'^2_i)^2\right)}{24A^2} \\ &\Rightarrow m = -h - x_a + \ln\left(\frac{A}{A-N}\right) + \frac{a^2\Sigma_i x'^2_i}{2A} - \frac{a^3\Sigma_i x'^3_i}{6A} \\ &+ \frac{a^4\left(A\Sigma_i x'^4_i - 3(\Sigma_i x'^2_i)^2\right)}{24A^2}\end{aligned} \tag{D.2}$$

A similar trick can be used to track the order of the terms in the expansion of $-\Sigma_{i=1}^{A}\ln\left[1+e^{(h+m+x_a+x_i')}\right]$, substituting in the expression for the Boltzmann factor. We are interested in the expansion of:

$$\begin{aligned}&\Sigma_{i=1}^{A}\ln\left[1+e^{(h+m+x_a+x_i')}\right]= \\ &= A\ln\left(e^c+1\right)+\frac{e^c a^2\Sigma_i x_i'^2}{2\left(e^c+1\right)^2} \\ &+\frac{e^c a^3\Sigma_i x_i'^3\left(1-e^c\right)}{6\left(e^c+1\right)^3}+\frac{e^c a^4\Sigma_i x_i'^4\left(e^{2c}-4e^c+1\right)}{24\left(e^c+1\right)^4}\ .\end{aligned} \tag{D.3}$$

Substituting in the expansion of the Boltzmann factor e^c, we find the series expansion of this term up to 4th order in a:

$$\begin{aligned}&-\Sigma_{i=1}^{A}\ln\left[1+e^{(h+m+x_a+x_i')}\right]=-A\ln\left(\tfrac{A}{A-N}+1\right) \\ &-\tfrac{a^2A\Sigma_i x_i'^2(3A-2N)}{2(N-2A)^2}+\tfrac{a^3A^2\Sigma_i x_i'^3(4A-3N)}{6(2A-N)^3} \\ &+\tfrac{a^4\left(3(\Sigma_i x_i'^2)^2\left(4A^3-5AN^2+2N^3\right)-A\Sigma_i x_i'^4\left(6A^3-8A^2N+5AN^2-2N^3\right)\right)}{24(N-2A)^4}\end{aligned} \tag{D.4}$$

Setting the series parameter $a=1$, we forget for the time being about the explicit dependency of the terms on A and N, and write:

$$\begin{aligned}&mN-\Sigma_{i=1}^{A}\ln\left[1+e^{(h+m+x_a+x_i')}\right]= \\ &= g_{0,II}(A,N,h)-\tfrac{g_{2,II}(A,N)}{2}\Sigma_i x_i'^2+\tfrac{g_{3,II}(A,N)}{3!}\Sigma_i x_i'^3 \\ &\quad+\tfrac{g_{4a,II}(A,N)}{4!}\Sigma_i x_i'^4+\tfrac{g_{4b,II}(A,N)}{4!}(\Sigma_i x_i'^2)^2\ .\end{aligned} \tag{D.5}$$

where we make the identifications:

$$\begin{aligned}g_{0,II}(A,N,h)&= N\ln\left(\tfrac{A}{A-N}\right)-A\ln\left(\tfrac{A}{A-N}+1\right)-h-x_a \\ g_{2,II}(A,N)&= \tfrac{A(3A-2N)}{(N-2A)^2}-\tfrac{N}{A} \\ g_{3,II}(A,N)&= \tfrac{A^2(4A-3N)}{(2A-N)^3}-\tfrac{N}{A} \\ g_{4a,II}(A,N)&= \tfrac{N}{A}-\tfrac{A\left(6A^3-8A^2N+5AN^2-2N^3\right)}{(N-2A)^4} \\ g_{4b,II}(A,N)&= \tfrac{3\left(4A^3-5AN^2+2N^3\right)}{(N-2A)^4}-\tfrac{3N}{A^2}\ .\end{aligned}$$

The action (or the effective free energy) can be re-expressed in terms of the average value of the Hubbard-Stratonovich field in order to substitute the above:

$$\begin{aligned}&\tilde{S_{II}}[\mathbf{x}',\mathbf{x}_a,A,N]= \\ &=\tfrac{1}{2}(\mathbf{x}_a+\mathbf{x}')^T\tilde{\mathbf{J}}'^{-1}(\mathbf{x}_a+\mathbf{x}')+g_{0,II}(A,N)-\tfrac{g_{2,II}(A,N)}{2}\Sigma_i x_i'^2 \\ &+\tfrac{g_{3,II}(A,N)}{3!}\Sigma_i x_i'^3-\tfrac{g_{4a,II}(A,N)}{4!}\Sigma_i x_i'^4+\tfrac{g_{4b,II}(A,N)}{4!}(\Sigma_i x_i'^2)^2\ .\end{aligned} \tag{D.6}$$

We will also need to check that the total contribution of the sixth order term is positive in order for the Hamiltonian to be bounded from below. Using the connection, Eqn. (B.9), between the Hubbard-Stratonovich field x and the density ϕ, we finbally obtain the action $S[\phi]$.

E. CONTINUOUS SENSOR CONCENTRATION

The partition function is given by the path integral of the action, given by Eqns. (C.6),(D.6) above, which plays the role of effective free energy in our thermally-equilibrated problem:

$$Z_\iota=\sqrt{\det\tilde{\mathbf{J}}'}\int D[\phi]e^{-S_{0,\iota}[\rho,A,N]-S_{1,\iota}[\phi,A,N]}$$

where the two parts of the action are given by Eqn. (??) after substituting Eqn. (B.9):

$$S_{0,\iota}[\rho_{\mathbf{a}},A,N]=A\beta J(\rho_a)^2+g_{0,\iota}(A,N) \tag{E.1}$$

$$\begin{aligned}S_{1,\iota}[\phi,A,N]&=\tfrac{\beta}{2}\Sigma_{ij}J_{ij}\phi_i\phi_j-\tfrac{\beta^2 g_{2,\iota}(A,N)}{2}\Sigma_i\left(\Sigma_j J_{ij}\phi_j\right)^2 \\ &+\tfrac{\beta^3 g_{3,\iota}(A,N)}{3!}\Sigma_i\left(\Sigma_j J_{ij}\phi_j\right)^3 \\ &+\tfrac{\beta^4 g_{4a,\iota}(A,N)}{4!}\Sigma_i\left(\Sigma_j J_{ij}\phi_j\right)^4+\tfrac{\beta^4 g_{4b,\iota}(A,N)}{4!}\left(\Sigma_i(\Sigma_j J_{ij}\phi_j)^2\right)^2\ ,\end{aligned} \tag{E.2}$$

where the couplings J_{ij} of the original theory have been restored, and the enumeration variable ι (hereafter not explicitly written except for clarity) indicates that the expression can refer to either Model I or II.

The order parameter can be transformed into wave vector space, imposing periodic boundary conditions:

$$\phi_i=\frac{1}{\sqrt{A}}\Sigma_{\mathbf{k}}e^{i\mathbf{k}\cdot\mathbf{s}_i}\phi'_{\mathbf{k}}\ , \tag{E.3}$$

where s_i is the position of the sensor i along the contact ring, and the wave vectors are quantised as $k_\mu=\frac{2\pi n_\mu}{N_\mu}$ with $n_\mu=0,1,...,N_\mu-1$ where N_μ is the number of lattice sites in the direction μ (this naturally allows the problem to be extended...) s.t. $\Pi_{\mu=1}^{D}N_\mu=A$ is the total number of lattice sites. Note that both real-space and reciprocal space variables are non-dimensional at this stage (as is the lattice size A), with the underlying natural length scale a defining the sensor size. We use the identity:

$$\frac{1}{A}\Sigma_i e^{i(\mathbf{k}-\mathbf{k}')\cdot\mathbf{r}_i}=\delta_{\mathbf{k},\mathbf{k}'} \tag{E.4}$$

to obtain the Fourier transform of the terms in the truncated effective action:

$$\begin{aligned}&\tfrac{\beta}{2}\Sigma_{ij}J_{ij}\phi_i\phi_j=\tfrac{\beta}{2}\Sigma_{\mathbf{k}}J_{\mathbf{k}}\phi_{-\mathbf{k}}\phi_{\mathbf{k}} \\ &\tfrac{\beta^2}{2}\Sigma_i\left[\Sigma_j J_{ij}\phi_j\right]^2=\tfrac{\beta^2}{2}\Sigma_{\mathbf{k}}J_{-\mathbf{k}}J_{\mathbf{k}}\phi_{-\mathbf{k}}\phi_{\mathbf{k}} \\ &\tfrac{\beta^3}{3!}\Sigma_i\left[\Sigma_j J_{ij}\phi_j\right]^3=\tfrac{\beta^3}{3!\sqrt{A}}\Sigma_{\mathbf{k_1},\mathbf{k_2}}\delta_{\mathbf{k}_1+\mathbf{k}_2+\mathbf{k}_3,0}J_{\mathbf{k}_1}J_{\mathbf{k}_2}J_{\mathbf{k}_3}\phi_{\mathbf{k}_1}\phi_{\mathbf{k}_2}\phi_{\mathbf{k}_3} \\ &\tfrac{\beta^4}{4!}\Sigma_i\left[\Sigma_j J_{ij}\phi_j\right]^4 \\ &=\tfrac{\beta^4}{4!A}\Sigma_{\mathbf{k}_1,\mathbf{k}_2,\mathbf{k}_3,\mathbf{k}_4}\delta_{\mathbf{k}_1+\mathbf{k}_2+\mathbf{k}_3+\mathbf{k}_4,0}J_{\mathbf{k}_1}J_{\mathbf{k}_2}J_{\mathbf{k}_3}J_{\mathbf{k}_4}\phi_{\mathbf{k}_1}\phi_{\mathbf{k}_2}\phi_{\mathbf{k}_3}\phi_{\mathbf{k}_4}\end{aligned}$$

where $J_{\mathbf{k}}$ is the Fourier transform of the exchange couplings $J_{ij}\equiv J(\mathbf{s}_i-\mathbf{s}_j)$:

$$J_{\mathbf{k}}=\Sigma_i e^{-i\mathbf{k}\cdot\mathbf{s}_i}J(\mathbf{r}_i)\ . \tag{E.5}$$

ϕ_i and J_{ij} are real, and the couplings are reciprocal: $J(-\mathbf{s})=J(\mathbf{s})$, so $\phi_{-\mathbf{k}}=\phi^*_{-\mathbf{k}}$ and $J_{-\mathbf{k}}=J_{\mathbf{k}}$.

Thus in Fourier space, the truncated effective action may be written:

$$\begin{aligned}S[\phi,A,N]&=\tfrac{\beta}{2}\Sigma_{\mathbf{k}}J_{\mathbf{k}}(1-\beta g_2(A,N)J_{\mathbf{k}})\phi_{-\mathbf{k}}\phi_{\mathbf{k}} \\ &+\tfrac{g_3(A,N)\beta^3}{3!\sqrt{A}}\Sigma_{\mathbf{k_1},\mathbf{k_2}}\delta_{\mathbf{k}_1+\mathbf{k}_2+\mathbf{k}_3,0}J_{\mathbf{k}_1}J_{\mathbf{k}_2}J_{\mathbf{k}_3}\phi_{\mathbf{k}_1}\phi_{\mathbf{k}_2}\phi_{\mathbf{k}_3} \\ &+\tfrac{g_{4a}(A,N)\beta^4}{4!A}\Sigma_{\mathbf{k}_1,\mathbf{k}_2,\mathbf{k}_3,\mathbf{k}_4}\delta_{\mathbf{k}_1+\mathbf{k}_2+\mathbf{k}_3+\mathbf{k}_4,0}J_{\mathbf{k}_1}J_{\mathbf{k}_2}J_{\mathbf{k}_3}J_{\mathbf{k}_4}\phi_{\mathbf{k}_1}\phi_{\mathbf{k}_2}\phi_{\mathbf{k}_3}\rho'_{\mathbf{k}_4} \\ &+\tfrac{g_{4b}(A,N)\beta^4}{4!}\left(\Sigma_{\mathbf{k}}J_{-\mathbf{k}}J_{\mathbf{k}}\phi_{-\mathbf{k}}\phi_{\mathbf{k}}\right)\left(\Sigma_{\mathbf{k}'}J_{-\mathbf{k}'}J_{\mathbf{k}'}\phi_{-\mathbf{k}'}\phi_{\mathbf{k}'}\right)+O(\phi_i^5)\ .\end{aligned} \tag{E.6}$$

Sufficiently close to the transition point, only long-range fluctuations (so $\mathbf{k}$ small) contribute significantly. So $J_{\mathbf{k}}$ can be expanded in powers of $\mathbf{k}$. If the coordination number is $z = 2D$, or explicitly $z = 4$ on our contact plane, then:

$$J_{\mathbf{k}} = J[z - \mathbf{k}^2] + O(k^4) \tag{E.7}$$

So the quadratic term in the field ρ' has the following coefficient:

$$\beta J_{\mathbf{k}}(1 - g_2\beta J_{\mathbf{k}}) = (r_0 + c_0\mathbf{k}^2) + O(k^4) \tag{E.8}$$

where $|T - T_c| \ll T_c$ is assumed (so the coefficient of the quadratic term in k simplifies) and the important constants r_0 and c_0 are defined as:

$$r_{0,\iota} = 4\beta J(1-4g_{2,\iota}\beta J) \ ; \quad c_{0,\iota} = 8g_{2,\iota}\beta^2 J^2 - \beta J \ . \tag{E.9}$$

The difference in the constants r_0 and c_0 between the two models arises from the difference between $g_{2,I}$ and $g_{2,II}$.

In the limit when the discrete set of allowed wave vectors merges into a continuum, we replace the momentum sums by integrations according to:

$$\frac{1}{V}\Sigma_{\mathbf{k}} \to \int \frac{d^D k}{(2\pi)^D} \equiv \int_{\mathbf{k}} \quad .$$

Normalising the fields by the continuum fields $\phi(\mathbf{k}) = a\sqrt{V}\phi'_{\mathbf{k}}$ instead, we define the coupling constants:

$$t_1 = g_3(A,N)a^{D-3}(\beta J_{\mathbf{k}=0})^3 \approx g_3(A,N)a^{D-3}\left(\tfrac{1}{g_2}\right)^3$$
$$u_1 = g_{4a}(A,N)a^{D-4}(\beta J_{\mathbf{k}=0})^4 \approx g_{4a}(A,N)a^{D-4}\left(\tfrac{1}{g_2}\right)^4$$
$$u_2 = g_{4b}(A,N)a^{-4}(\beta J_{\mathbf{k}=0})^4 \approx g_{4b}(A,N)a^{-4}\left(\tfrac{1}{g_2}\right)^4 = \frac{g_{4b}(A,N)}{g_{4a}(A,N)a^D}u_1 \quad .$$

In the main text, we treat the evolution of a sensor concentration on the adhesion contact plane between a cell and a surface as a $2D$ problem. It is clear that $D = 2$, if we set $V = A$, and set the inter-site spacing to be 1 (scaling all lengths by a), then the above constants simplify considerably. Wavevectors should also be rewritten without any confusion in scalar form.

To check the dimensionality, remember that in this situation, there are in effect not four but three integrals, as one is eliminated in the evaluation of the delta function. The effective action becomes (note, there is an ultraviolet cut-off to the integration, hence the subscript Λ_0):

$$\begin{aligned} S_{\Lambda_0}[\phi, A, N] &= \tfrac{1}{2}\textstyle\int_k [r_0 + c_0 k^2]\phi(-k)\phi(k) \\ &+ \tfrac{t_1}{3!}\textstyle\int_{k_1}\int_{k_2}\int_{k_3}(2\pi)\delta(k_1+k_2+k_3)\phi(k_1)\phi(k_2)\phi(k_3) \\ &+ \tfrac{u_1}{4!}\textstyle\int_{k_1}\int_{k_2}\int_{k_3}\int_{k_4}(2\pi)\delta(k_1+k_2+k_3+k_4)\phi(k_1)\phi(k_2)\phi(k_3)\phi(k_4) \\ &+ \tfrac{u_2}{4!}\left(\textstyle\int_k \phi(-k)\phi(k)\right)\left(\textstyle\int_{k'} \phi(-k')\phi(k')\right) + O((\phi)^5) \quad . \end{aligned} \tag{E.10}$$

Transforming into real space, we obtain the effective free energy for the continuous variable $\phi(r)$ and its gradients:

$$\begin{aligned} S_{\Lambda_0}[\phi, A, N] = &\textstyle\int_0^A ds\Big[\tfrac{r_0}{2}\phi^2(s) + \tfrac{c_0}{2}[\nabla\phi(s)]^2 + \tfrac{t_1}{3!}\phi^3(s) \\ &+ \tfrac{u_1}{4!}\phi^4(s) + \tfrac{u_2}{4!}\phi^2(s)\Big(\textstyle\int d^D s' \phi^2(s')\Big)\Big] \ . \end{aligned} \tag{E.11}$$

F. BEHAVIOUR AWAY FROM THE TRANSITION POINT

The action in Eqn.(E.11) is hard to analyse without making some simplifying assumptions. We saw in the previous section that fluctuations in the sensor concentration are well-described by a set of sinusoidal perturbations with a given wavenumber k near the transition point. We can examine each of these contributions separately while fluctuations are still sinusoidal (*i.e.* provided that the cubic, quartic and higher order terms in the action do not dominate over the quadratic term), considering:

$$\begin{aligned} \phi_k(s,t) &= A_k(0)e^{\frac{k^2}{4}\left(-r_0 - \frac{c_0 k^2}{4}\right)t}\cos\left(\frac{ks}{2}\right) \\ &= \kappa_k(t)\cos\left(\frac{ks}{2}\right) \quad , \end{aligned} \tag{F.1}$$

where we continue using the non-dimensional lengths scaled by the sensor soze a. In the long-time limit, a fastest growing mode will dominate, and so we will approximately find that:

$$\begin{aligned} \phi(r,t) &= \Sigma_k \phi_k(s,t) \approx \phi_{k_{\max}}(s,t) \\ &\approx \kappa_{k_{\max}}(t)\cos\left(\frac{k_{\max}s}{2}\right) \approx \phi_{\max}\cos\left(\frac{k_{\max}s}{2}\right) \end{aligned} \tag{F.2}$$

where $\phi_{\max}$ is the amplitude of fluctuations. This enormously simplifies the problem, as the integral in the fourth order term can be approximated as:

$$\textstyle\int ds'(\phi(s',t))^2 \approx (\phi_{\max})^2 \int ds' \cos^2\left(\frac{k_{\max}s}{2}\right) \approx \frac{A}{2}(\phi_{\max})^2$$

The Ginzburg-Landau action can then be written in this limit as:

$$\begin{aligned} S_{\Lambda_0}[\phi] = \textstyle\int ds\Big[&\tfrac{r_0}{2}\phi^2_{\max}\cos^2\left(\tfrac{k_{\max}s}{2}\right) + \tfrac{c_0}{2}\phi^2_{\max}\sin^2\left(\tfrac{k_{\max}s}{2}\right) \\ &+ \tfrac{t_1}{3!}\phi^3_{\max}\cos^3\left(\tfrac{k_{\max}s}{2}\right)\Big] \\ &+ \tfrac{u_1}{4!}\phi^4_{\max}\cos^4\left(\tfrac{k_{\max}s}{2}\right) + \tfrac{A}{2}\tfrac{u_2}{4!}\phi^4_{\max}\cos^2\left(\tfrac{k_{\max}s}{2}\right)\Big] \ . \end{aligned} \tag{F.3}$$

Now $\cos^2\theta \geq \cos^4\theta, \forall\theta$, so if we show that $u_2 > 0$ and that the fourth order terms in the action above are positive for $k_{\max}s = 2m\pi$, for integer m, then they will be positive at every point s along the boundary of the cell.

Using the results from part C and D, we can show that when $k_{\max}s = 2m\pi$, the fourth order terms in both Models combine to:

$$4^{\text{th}} \text{ order terms, model I } = \tfrac{b^5+3b^4-2b^3+b}{12(b+1)^4(4!g_2^4)}\phi^4_{\max} \tag{F.4}$$

and

$$4^{\text{th}} \text{ order terms, model II } = \tfrac{-b^5+8b^4-14b^3+7b^2+2b}{2(2-b)^4(4!g_2^4)}\phi^4_{\max}$$

where $b = N/A$. These combinations are both positive $\forall N < A$, so we expect the fourth order terms to be positive for both Models at all positions r along the

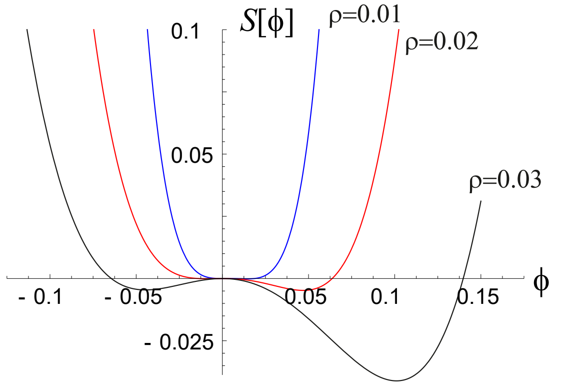

FIG. S1. Plot of the Ginzburg-Landau action as a function of the amplitude of density fluctuation as the average cadherin density increases past the transition value for the gas-liquid phase transition for Model I. Cadherin nearest-neighbour interactions are taken to be $J = 5k_BT$ (see main text). As the area diminishes, $\rho = N/A$ increases and the $\phi = 0$ configuration (average concentration) changes from a global minimum to a local maximum of the action, and two minima develop on either side of the average sensor concentration; the system undergoes a phase transition from a uniform sensor distribution to a two phase distribution in which a denser and a more dilute phase coexist. The profile is asymmetric due to the presence of a non-zero third order term. This term is small and it is possible that the asymmetry in the action might be attributable to the error in the Boltzmann factor approximation introduced in Eqn. (B.13) as the condition that $N - A \ll A$ is no longer verified for smaller values of N/A.

cell boundary, for all sensor concentrations for which our Boltzmann factor approximation is appropriate (so $A - N \ll A$) and when the modes have grown for a sufficiently long time that the mode closest to the fastest growing spatial frequency $k_{\max}$ dominates.

In this limit, a fourth order expansion of the Ginzburg-Landau action is therefore sufficient to describe the destabilization and initial growth of spatial modes (by analysing the quadratic terms) as well as the development of two slightly asymmetrical minima in the action profile (due to the presence of non-zero third order terms) relative to the cell-wide average sensor concentration. This behaviour is examined in Fig. S1.

G. FASTEST GROWING MODE: MODEL I, LOW CONCENTRATION

We use the results in Eqn. (7) of the main text to determine the fastest growing wavenumber as a function of the radius of the cell, for a fixed total number of sensors N and interaction energy J. We substitute the fraction of occupied sensor sites $b = N/A$ instead of N and A, and use the scaled interaction strength $\beta J = j$ as a shorthand. Substituting these values into the expression for the fastest growing mode number, Eqn. (13) of the main text, we find:

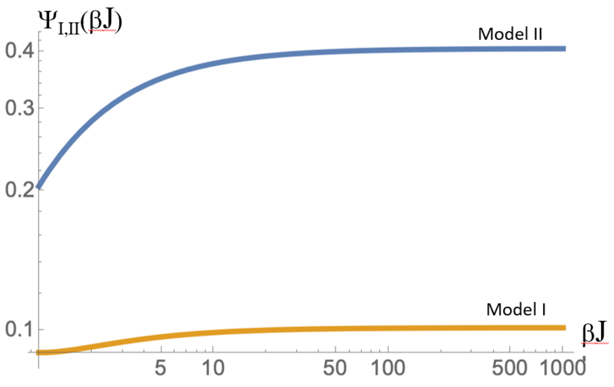

FIG. S2. Log-log plots of $\Psi_I(j)$ and $\Psi_{II}(j)$ for Models I and II, showing that it is becomes quite constant for $j > 10$.

$$\|\mathbf{k}\|_{\max} = \sqrt{2}\sqrt{-r_{0,I}/c_{0,I}} \tag{G.1}$$
$$= 2\sqrt{2}\sqrt{\frac{\left(4b\left(b^2+b+1\right)j - (b+1)^2\right)}{8b\left(b^2+b+1\right)j - (b+1)^2}}\ .$$

Next we solve for the transition point where $r_0 = 0$. This gives us the value $b = b_0$, around which we perform our series expansion:

$$b_0(j) = \frac{1}{12j}\Big(-\zeta(j) + (1-4j) + \frac{32j^2 - 16j - 1}{\zeta(j)}\Big)$$

where we use the shorthand:

$$\zeta(j) = \sqrt[3]{24\sqrt{3}\sqrt{48j^6 - 16j^5 + 20j^4 + j^3} - 224j^3 - 48j^2 - 24j - 1}$$

Inserting $j = \beta J = 7$, we find $b_0 = N/A = 0.01$. The series expansion is asymmetrical: the square root in Eqn. H.1 is only defined when $b > b_0$. We convert the wavevector into mode numbers $m_x/L_x \approx m_y/L_y \approx (a\,\|\mathbf{k}\|_{\max})/(2\sqrt{2}\pi)$ and find a series expansion for the total number of clusters $M = m_x m_y$ around the transition point as:

$$M_{\max} = Aj\Psi_I(j)\left(\frac{b}{b_0(j)} - 1\right) + O((b_0 - b)^2) \tag{G.2}$$

where the coefficient $\Psi_I(j)$ takes the messy form:

$$\Psi_I(j) = \frac{4b_0\left(b_0^4 + 4b_0^3 + 4b_0^2 + 2b_0 + 1\right)}{\pi^2\left(8b_0^3 j + 8b_0^2 j - b_0^2 + 8b_0 j - 2b_0 - 1\right)^2}\ .$$

b_0 itself depends strongly on j, so it is not immediately obvious how $\Psi_I(j)$ will depend on j, but we find in Fig. S2 that it is quite constant above $j \approx 10$.

In Fig. S3, we show a comparison between the series-approximated expression and the exact expression for the number density of clusters at the transition, for $A = 20000$ ($1\mu m^2$ lattice) and $\beta J = 5$. This means that the series expression is good for small changes in contact area (up to $5-10\%$) away from the transition point.

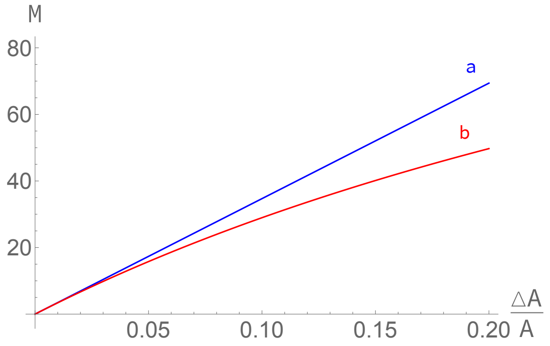

FIG. S3. Comparison between the series approximation for the number density of clusters (curve a) to the real value of the number density of clusters at the phase transition for $\beta J = 11$ (curve b). For small changes in $(A_0 - A)/A$, of the order of up to $5-10\%$, the series approximation is good. We find in the main text that cadherin clusters aggregate at a number density $M^* \approx 30$, so the series approximation for m is good for our purposes.

Finally, we need to re-express Eqn. (H.2) into a more useful form for the reasoning in the main text, which leads to the estimate of the time to destabilize the uniform density field $\rho = N/A$. Instead of using the ratio $b = \langle \rho \rangle = N/A$, we express m in terms of changes in the size of the lattice $\Delta A = A_0 - A = A(b/b_0 - 1)$. The ratio of mechanosensors to lattice sites only depends on the scaled interaction energy $j = \beta J$, so we write:

$$M_{\max} = j\Psi_I(j)\Delta A + O((b_0 - b)^2) \ . \tag{G.3}$$

The experimentally useful value is not the change in the dimensionless lattice size, but rather of the area of the cell. To convert to correctly dimensional quantities, we see that $\Delta A = t_1 \dot{\Sigma}_{\text{shrink}}/a^2$, where a is the distance between lattice sites (7nm). We find that the number of adhesions corresponding to the fastest growing mode number $M(t_1)$ depends on the time elapsed since contact area passed the transition point t_1:

$$M(t_1) \approx \beta J \Psi_I(\beta J) \left(\frac{t_1 \dot{\Sigma}_{\text{shrink}}}{a^2} \right) \ . \tag{G.4}$$

H. FASTEST GROWING MODE: MODEL II, HIGH CONCENTRATION

As in the previous section, we use the expression for the fastest growing mode number, Eqn. (13) of the main text, to find:

$$\begin{aligned} \|\mathbf{k}\|_{\max} &= \sqrt{2}\sqrt{-r_{0,II}/c_{0,II}} \qquad\qquad (\text{H.1}) \\ &= 2\sqrt{2}\sqrt{\frac{4(b-1)((b-3)b+3)j+(b-2)^2}{8(b-1)((b-3)b+3)j+(b-2)^2}} \ . \end{aligned}$$

Next we solve for the transition point where $r_0 = 0$. This gives us the value $b = b_0$, around which we perform our series expansion:

$$b_0(j) = \frac{1}{12j}\left(\zeta(j) + (16j-1) + \frac{1+16j-32j^2}{\zeta(j)}\right)$$

with the same shorthand as above:

$$\zeta(j) = \sqrt[3]{24\sqrt{3}\sqrt{48j^6 - 16j^5 + 20j^4 + j^3} - 224j^3 - 48j^2 - 24j - 1}$$

Inserting $j = \beta J = 11$, we find $b_0 = N/A = 0.977$. The series expansion is asymmetrical: the square root in Eqn. H.1 is only defined when $b < b_0$. We convert the wavevector into mode numbers $m_x/L_x \approx m_y/L_y \approx (a\,\|\mathbf{k}\|_{\max})/(2\sqrt{2}\pi)$ and find a series expansion for the total number of clusters $M = m_x m_y$ around the transition point as:

$$M_{\max} = Aj\Psi_{II}(j)\left(1 - \frac{b}{b_0(j)}\right) + O((b_0 - b)^2) \tag{H.2}$$

where the coefficient $\Psi_{II}(j)$ takes the messy form:

$$\Psi_{II}(j) = \frac{4b_0\left(b_0^4 - 8b_0^3 + 22b_0^2 - 26b_0 + 12\right)}{\pi^2\left(8b_0^3 j - 32b_0^2 j + b_0^2 + 48b_0 j - 4b_0 - 24j + 4\right)^2}$$

and is plotted in Fig. S2. As in the previous section (see Fig. S3), this series expression is also good for small changes in contact area away from the transition point.

Once again, Eqn. (H.2) is better experessed in terms of changes in the size of the lattice $\Delta A = A - A_0 = A(1 - b/b_0)$. As in the previous section, we find that the ratio of mechanosensors to lattice sites only depends on the scaled interaction energy $j = \beta J_C$ (J_C is the cadherin-p120 interaction energy $11.0k_BT$). The maximum number of clusters for the lattice is:

$$M_{\max} = j\Psi_{II}(j)\Delta A + O((b_0 - b)^2) \ . \tag{H.3}$$

Converting once more to correctly dimensional quantities, $\Delta A = t_1 \dot{\Sigma}_{\text{grow}}/a_C^2$, where a_C is the distance between lattice sites (17.2nm). The number of adhesions corresponding to the fastest growing mode number $M(t_1)$depends on the time elapsed since the contact area passed the transition point t_1:

$$M(t_1) \approx \beta J \Psi_{II}(\beta J) \left(\frac{t_1 \dot{\Sigma}_{\text{grow}}}{a^2} \right) \ . \tag{H.4}$$

[1] S. F. Fenz, T. Bihr, D. Schmidt, R. Merkel, U. Seifert, K. Sengupta, and A.-S. Smith, Nat. Physics **13**, 906 (2017).

[2] M. Dembo, D. C. Torney, K. Saxman, and D. Hammer, Proceedings of the Royal Society B: Biological Sciences **234**, 10.1098/rspb.1988.0038 (1988).

[3] G. I. Bell, Science **200**, 10.1126/science.347575 (1978).

[4] S. Bajpai, J. Correia, Y. Feng, J. Figueiredo, S. X. Sun, G. D. Longmore, G. Suriano, and D. Wirtz, Proceedings of the National Academy of Sciences of the United States of America **105**, 18331 (2008).

[5] D. Fichtner, B. Lorenz, S. Engin, C. Deichmann, M. Oelkers, A. Janshoff, A. Menke, D. Wedlich, and C. M. Franz, PLoS One **9**, e93123 (2014).

[6] A. Biswas, A. Alex, and B. Sinha, Biophysical Journal **113**, 10.1016/j.bpj.2017.08.041 (2017).

[7] M. C. D. Santos, R. Déturche, C. Vézy, and R. Jaffiol, Biophysical Journal **111**, 10.1016/j.bpj.2016.06.043 (2016).
